# Pathogenetic Significance of Fetal-Type Acetylcholine Receptors on Thymic Myoid Cells in Myasthenia Gravis

**DOI:** 10.1155/1992/40576

**Published:** 1992

**Authors:** Kerstin I. Geuder, Alexander Marx, Veit Witzemann, Berthold Schalke, Klaus Toyka, Thomas Kirchner, Hans-Konrad Müller-Hermelink

**Affiliations:** 1 Pathologisches Institut and Neurologische Klinik Universität Würzburg Germany; 2 Pathologisches Institut der Universität Josef Schneider Str. 2 Würzburg 8700 Germany; 3 Max Planck Institut für Medizinische Forschung Heidelberg Germany

**Keywords:** Myasthenia, thymus, myoid cell, acetylcholine receptor, muscle development, autoimmunity

## Abstract

To investigate the role of thymic myoid cells in the pathogenesis of myasthenia gravis (MG), mRNA of nonneoplastic thymuses from eight MG patients was analyzed by dot blot hybridization for the occurrence of acetylcholine receptor (AChR) subunit transcripts, using the five AChR-subunit cDNAs (alpha, beta, gamma, delta, and epsilon) as probes. Attention was particularly paid to the gamma- and epsilon-subunit transcripts that specify fetal- or adult-type AChR. In all eight thymuses, transcripts of the alpha-, beta-, gamma-, and delta-subunit genes were detected. Relative autoradiographic signal intensities correlated with the frequencies of thymic myoid cells as determined by immunostaining with anti-AChR monoclonal antibodies. In only one of these thymuses were transcripts of the epsilon-subunit gene detected in addition to those of the other subunit genes. Four MG-associated thymomas without myoid cells were devoid of any AChR-subunit mRNA. Our findings imply that fetal-type AChR is expressed in MG thymuses as a rule, whereas adult-type AChR is coexpressed with it only in a minority of cases. A similar pattern of cotranscription is known to occur at certain stages of muscle development, and can be found in human rhabdomyosarcomas with an intermediate stage of myogenesis. Because the serum autoantibodies of MG patients exhibit preferential reactivity with fetal AChRs, the presence of fetal AChRs in the thymus provides circumstantial evidence for an active involvement of thymic myoid cells in the autoimmune process.


*Developmental Immunology*, 1992, Vol. 2, pp. 69–75
Reprints available directly from the publisher
Photocopying permitted by license only

© 1992 Harwood Academic Publishers GmbH
Printed in the United Kingdom

### Pathogenetic Significance of Fetal-Type Acetylcholine Receptors on Thymic Myoid Cells in Myasthenia Gravis

KERSTIN I. GEUDER,*† ALEXANDER MARX,† VEIT WITZEMANN,‡ BERTHOLD SCHALKE,† KLAUS TOYKA,† THOMAS KIRCHNER,† and HANS-KONRAD MÜLLER-HERMELINK†

*†Pathologisches Institut and Neurologische Klinik, Universität Würzburg, Germany*

*‡Max Planck Institut für Medizinische Forschung, Heidelberg, Germany*

To investigate the role of thymic myoid cells in the pathogenesis of myasthenia gravis (MG), mRNA of nonneoplastic thymuses from eight MG patients was analyzed by dot blot hybridization for the occurrence of acetylcholine receptor (AChR) subunit transcripts, using the five AChR-subunit cDNAs (alpha, beta, gamma, delta, and epsilon) as probes. Attention was particularly paid to the gamma- and epsilon-subunit transcripts that specify fetal- or adult-type AChR. In all eight thymuses, transcripts of the alpha-, beta-, gamma-, and delta-subunit genes were detected. Relative autoradiographic signal intensities correlated with the frequencies of thymic myoid cells as determined by immunostaining with anti-AChR monoclonal antibodies. In only one of these thymuses were transcripts of the epsilon-subunit gene detected in addition to those of the other subunit genes. Four MG-associated thymomas without myoid cells were devoid of any AChR-subunit mRNA. Our findings imply that fetal-type AChR is expressed in MG thymuses as a rule, whereas adult-type AChR is coexpressed with it only in a minority of cases. A similar pattern of cotranscription is known to occur at certain stages of muscle development, and can be found in human rhabdomyosarcomas with an intermediate stage of myogenesis. Because the serum autoantibodies of MG patients exhibit preferential reactivity with fetal AChRs, the presence of fetal AChRs in the thymus provides circumstantial evidence for an active involvement of thymic myoid cells in the autoimmune process.

KEYWORDS: Myasthenia, thymus, myoid cell, acetylcholine receptor, muscle development, autoimmunity.

### INTRODUCTION

Myasthenia gravis (MG) is an autoimmune disease characterized by abnormally fatiguable neuromuscular transmission caused in 90% of patients by heterogeneous autoantibodies against the nicotinic acetylcholine receptors (AChR) of muscle endplates that are either reduced or functionally blocked (Compston et al., 1980; Drachman et al., 1980; Vincent et al., 1987). Heterogeneous thymic alterations are another feature of MG: thymitis—with or without lymphoid follicular hyperplasia—occurs in about 70% of patients, and thymic atrophy and thymoma are present in 20% and 10%, respectively (Kirchner et al., 1986; Müller-Hermelink et al., 1986). The relationship between these thymic abnormalities and the mechanisms of tolerance breakdown have not been fully established, but seem to be different (Compston et al., 1980; Chilosi et al., 1986). Although there is now strong evidence that tumor proteins with only a limited molecular similarity to the AChR may play a causative role in the pathogenesis of thymoma-associated MG (Kirchner et al., 1987, 1988; Marx et al., 1989, 1990; Geuder et al., 1989; Siara et al., 1991), the myoid cells of the thymic medulla have been suggested to be involved in autosensitization in non-thymoma-associated cases (Van de Velde et al., 1966; Kao and Drachman, 1977; Wekerle and Ketelsen, 1977). In fact, myoid cells were shown to express AChR (Schluep et al., 1987; Kirchner et al., 1988; Marx et al., 1992). However, it is controversial whether these AChR are only targets of anti-AChR autoantibodies (Schluep et al., 1987) or whether they are also involved in the trig-

*Corresponding author: Present address: Pathologisches Institut der Universität, Josef Schneider Str. 2, 8700 Würzburg, Germany.

TABLE 1

Summary of Results

| Thymus | α | β | δ | ε | γ Expr. |
|---|---|---|---|---|---|
| 4894/87 | + | + | + | – | + |
| 31252/87 | + | + | + | – | + |
| 27306/87 | + | + | + | – | + |
| 10209/89 | + | + | + | – | + |
| 18449/89 | + | + | + | – | + |
| 6983/89 | + | + | + | + | + |
| 1574/90 | + | + | + | – | + |
| 11199/90 | + | + | + | – | + |

Identification of transcripts for the AChR alpha, beta, gamma, delta, and epsilon subunit in MG thymuses by dot blot analysis of thymus mRNA with the respective cDNAs as probes. In Contrast to Fig. 2, only the presence or absence of a respective mRNA is indicated but not relative signal intensities.

gering or maintainance of autoantibody production (Kirchner et al., 1988).

The AChR of skeletal muscle is a pentameric cation channel composed of four different subunits (Changeux and Revah, 1987; Kubalek et al., 1987; Dipaola et al., 1989). Athough alpha, beta, and delta subunits are indispensable parts of fully functional AChR, the additional presence of either the gamma or the epsilon subunit determines the fetal or adult receptor type, respectively (Mishina et al., 1986). The fetal type of AChR is not only present during development, but is also reexpressed in adult muscles after denervation (Witzemann et al., 1987) and is referred to as extrajunctional AChR. Only at prenatal developmental stages are fetal and adult AChRs physiologically coexpressed in appreciable amounts (Mishina et al., 1986). In contrast, the adult AChR is the only detectable receptor type in postnatal innervated muscle, where it is confined to the endplates (Witzemann et al., 1989; Brenner et al., 1990).

Thymic myoid cells are the only differentiated muscle cells in adults presumed not to be physiologically innervated. However, whether they express adult in addition to the demonstrated extrajunctional type AChR (Schleup et al., 1987) is not clear. To address this question, we have probed thymus mRNA with the AChR alpha-, beta-, gamma-, delta, and epsilon-subunit cDNAs. Given the known preferential reactivity of anti-AChR autoantibodies with extrajunctional AChR (Weinstein and Hall, 1980; Vernet-der Garabedian et al., 1988), our finding of transcripts coding for the fetal AChR supports the view that thymic myoid cells participate actively in the pathogenesis of MG.

### RESULTS

### Human Thymuses Contain Transcripts for a Fetal-Type AChR

As summarized in Table 1, all eight MG thymuses investigated exhibited transcription of the

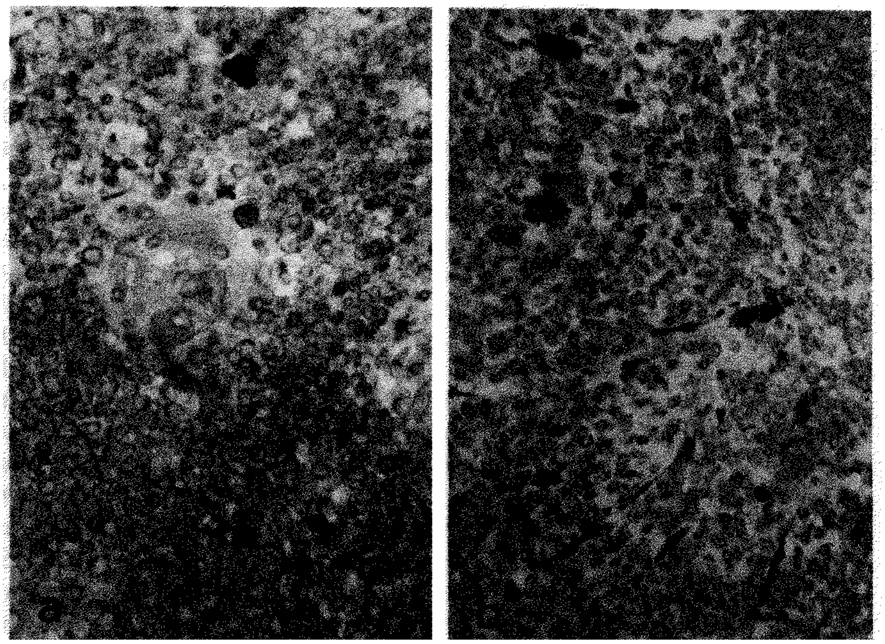

FIGURE 1. Immunohistochemical identification of thymic myoid cells in the thymic medulla by the anti-AChR monoclonal antibody mAb155 (Tzartos et al., 1986). For correlation with autoradiographic intensities of dot blots (see Fig. 2), the relative number of thymic myoid cells was semiquantitatively assessed (prior to mRNA extraction) by two independent observers as either few (not shown), some (a) or many (b).

| Thymus Histology | Autoradiograph | Relative Number of Thymic Myoid Cells |
|---|---|---|
| LFH |  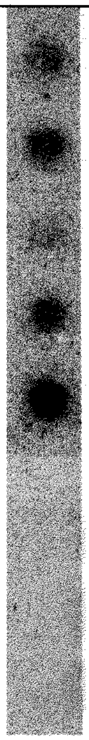 | few |
| Normal | | some |
| LFH | | few |
| LFH | | some |
| LFH | | many |
| Thymoma | | none |
| Thymoma | | none |
| Thymoma | | none |
| Thymoma | | none |

LFH = Lymphoid Follicular Hyperplasia

FIGURE 2. Approximate correlation between the relative number of thymic myoid cells and the signal intensities of autoradiographs of thymus mRNA dot blots probed with the AChR alpha-subunit cDNA. Extraction of mRNA from five thymuses, blotting (on the same filter), and autoradiography were done simultaneously and strictly in parallel to make signal intensities comparable.

alpha, beta, gamma, and delta AChR subunit genes as detected by mRNA dot blotting, using the respective AChR subunit cDNAs as probes. If concomitantly present within a given cell, these transcripts give rise to the expression of fetal AChR (Mishina et al., 1986). The specificity of the blotting procedure was checked in two ways. First, a distinct correlation was encountered between the frequencies of thymic myoid cells as determined by immunohistochemistry (Fig. 1) and the relative intensities of autoradiographs of the respective dot blots probed with the AChR alpha-subunit cDNA (Fig. 2). For this experiment, mRNA was extracted from five thymuses simultaneously and strictly in parallel. Second, the specificity of hybridization was determined by including mRNA from MG-associated thymomas in which myoid cells were absent (Fig. 2), or the carcinoma cell line A431 (Giard et al., 1973) as negative controls (Fig. 3; Geuder et al., 1989, 1992). Finally, the absence of any hybridization of the AChR gamma-subunit cDNA with mRNA from innervated adult human skeletal muscle (Fig. 4) confirms the specificity of our method.

### A Minority of Human Thymuses Contains Transcripts for Adult AChR

In addition to the transcripts for fetal AChR, only one out of eight thymuses exhibited transcription of the AChR epsilon-subunit gene (Table 1 and Fig. 3) indicating the potential to express adult/junctional-type AChR in addition to fetal AChR (Mishina et al., 1986). Interestingly, this case (#6982/89) showed a severe cortical involution after a corticosteroid treatment, which was not performed in the other patients.

### DISCUSSION

In the present investigation, we have shown that as a rule, thymuses of MG patients contain transcripts for fetal-type AChR. This finding is in

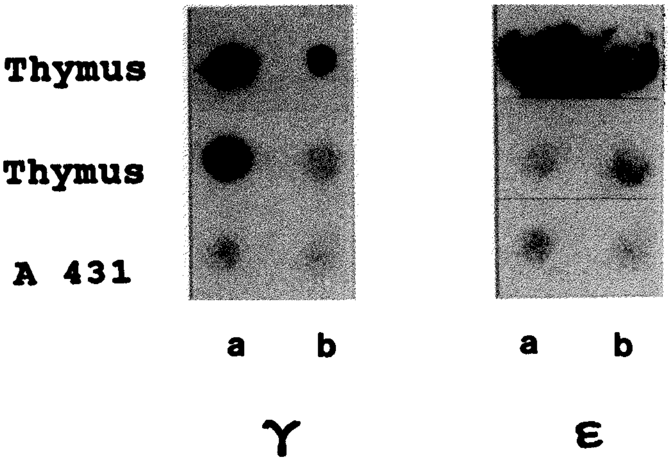

FIGURE 3. Cotranscription of the AChR gamma- and epsilon subunit gene in an MG thymus (#6983/89) as determined by mRNA dot blots probed with the respective cDNA. Positivity in #6983/89 for epsilon message is visible in the 5-$\mu$g dot that is not overloaded as is the 10-$\mu$g dot. For comparison, an MG thymus is shown that has only AChR gamma-subunit transcripts (#11199/90). mRNA from the human carcinoma cell line A431 served as negative control. a=10-$\mu$g, b=5-$\mu$g mRNA.

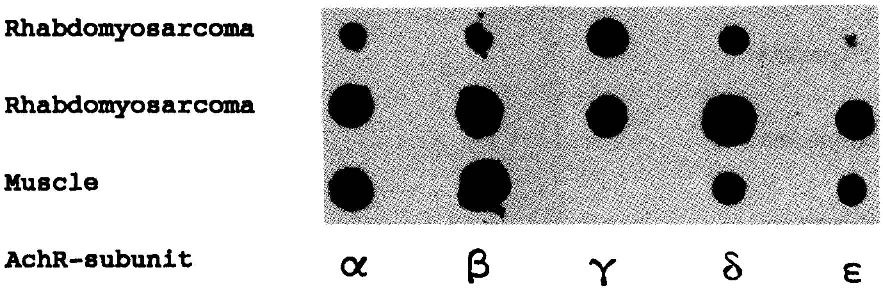

FIGURE 4. Cotranscription of all five AChR subunit genes within a tissue is not unique to thymic myoid cells, but is also encountered in human rhabdomyosarcomas (a, b), and is physiologically present during the perinatal period in developing muscle (not shown; Mishina et al., 1986). In contrast, adult human muscle exhibits transcription only of the alpha-, beta-, delta-, and epsilon-gene coding for the adult/junctional type of AChR (c).

keeping with previous immunohistochemical investigations that applied a monoclonal antibody specific for the human extra-junctional AChR type (Schluep et al., 1987). In adult bovine thymuses, the expression of fetal AChR as the only AChR type of myoid cells was also shown immunohistochemically by gamma- and epsilon-subunit specific mAbs (Nelson and Conti-Tronconi, 1990). Our additional investigations also demonstrated transcripts exclusively of the fetal AChR in normal thymuses containing myoid cells (in preparation). Using dot blotting, we did not detect transcripts of the AChR in MG-associated thymomas without myoid cells (Fig. 2), whereas Hara et al. (1991) recently reported a positivity by applying the PCR method.

We also show here that in a minority of human thymuses, epsilon-subunit mRNA, which characterizes the adult-type receptor, is present in addition to the other subunit mRNAs (Fig. 3). Whether this cotranscription correlates to a corticosteroid pretreatment of MG patients (as in our case) has to be proven by further studies (in preparation). The same finding of transcription of all five AChR subunit genes was recently reported in a rat thymus myoid cell line (Kobayashi et al., 1990). However, without *in situ* hybridization, we cannot be certain whether the mRNAs for all five AChR subunits occur together in single myoid cells, or if these myoid cells only express either fetal- or adult-type receptor. We cannot even exclude transcription of single AChR subunit genes in individual cells that would not result in any functional receptor (Mishina et al., 1986).

Adult and fetal AChR may cooccur within the same muscle during a short pre- and postnatal period (Mishina et al., 1986; Witzmann et al., 1989; Brenner et al., 1990). In addition, we have identified transcripts for both AChR types in rhabdomyosarcomas and in nephroblastomas that exhibit a partial rhabdomyomatous differentiation (Kirchner et al., 1990). As there is presumably no innervation in either tumors or thymic myoid cells, innervation is evidently not the only signal (Brenner et al., 1990) that initiates transcription of the AChR epsilon-subunit gene.

The present finding of fetal-type AChR mRNA in all MG thymuses investigated has implications for the role of thymic myoid cells in the pathogenesis of MG. On the one hand, thymic myoid cells are the only differentiated muscle cells in adults that are not innervated physiologically and that are therefore expected to express fetal-type AChR. On the other hand, extrajunctional/fetal AChRs are preferentially precipitated by MG sera (Weinstein and Hall, 1980; Verent-der Garabedian et al., 1988). We therefore conclude that AChRs on thymic myoid cells are not just targets of anti-AChR autoantibodies, but are antigens that actively participate in the stimulation of AChR-specific T and B cells. This might be confirmed if the autoantibodies to fetal AChR prove to precede those to endplate receptors during the course of MG.

### MATERIALS AND METHODS

### Patients and Tissues

Eight thymuses and four thymomas (as controls) were obtained from myasthenia gravis patients within 15 min after surgery and snap frozen in liquid nitrogen. Thymitis was classified according to Kirchner et al. (1986) according to the degree of lymphoid follicular hyperplasia or diffuse B-cell infiltration.

The diagnosis of myasthenia gravis was based on clinical findings, including electrophysiological investigations and the detection of serum anti-AChR autoantibodies (Table 2).

### Methods

Immunohistochemistry was performed on cryostat sections of snap frozen tissues applying a three-stage immunoperoxidase technique as described in detail previously (Kirchner et al., 1988). Thymic myoid cells were identified by culture supernatant of monoclonal antibody mAb155 directed against the cytoplasmic epitope alpha371-378 of the AChR alpha subunit (Tzartos et al., 1986) and by a purified monoclonal antidesmin antibody (Laboserv, Giessen, FRG).

Extraction of RNA, preparation of Poly(A)$^+$RNA by oligo-dT chromatography, and hybridization procedures under high stringency followed standard protocols (Ausubel et al., 1987). Filters were hybridized at 42 °C for 12 hr and washed at 55 °C twice for 30 min each with 1 ×SSC/2% SDS and then for 15 min with 0.1× SSC/0.2% SDS. The probes were cDNAs of the human AChR alpha and delta subunit (Schoepfer et al., 1988; Luther et al., 1989), the mouse AChR beta and gamma subunit (Boulter et al., 1985; Patrick et al., 1987), and the rat AChR epsilon subunit (Witzemann et al., 1990). Probes were $^{32}$P-labeled to a specific activity of $10^9$ dpm/$\mu$g DNA using a random primer DNA labeling protocol (Boehringer, Mannheim, Germany).

TABLE 2
Clinical and Pathological Findings in the Patients Included in This Study

| Case No. | Age (y) | Sex | Disease stage (Osserman, 1958) | Serum antibodies against AChR[a] | Disease duration before thymectomy (y) | HLA-Haplotype A | HLA-Haplotype B | DR | Thymus histology |
|---|---|---|---|---|---|---|---|---|---|
| 27306/87 | 34 | f | IIa | ++ | 2 | 1/2 | 8/39 | 2/3 | LFH[c] |
| 4894/87 | 15 | f | IIb | −/+ | 1 | 2/28 | 14/44 | 5 | Normal |
| 31252/87 | 9 | f | IIb | + | 0.75 | 1/3 | 8/55 | 1/3 | LFH |
| 31611/87 | 27 | f | IIa | ++ | 4 | 1/11 | 35/44 | 4/11 | LFH |
| 18449/89 | 11 | f | IIb | + | 0.3 | 1/2 | 8/44 | 3 | LFH |
| 6983/89 | 8 | f | III | −/+ | 0.5 | n.k.[b] | n.k. | n.k. | LFH[d] |
| 10209/89 | 61 | f | IIb | − | 0.6 | 2/24 | 44/60 | 4/5 | LFH |
| 1574/90 | 24 | f | IIa | ++ | 5 | n.k. | n.k. | n.k. | LFH |
| 11199/90 | 24 | f | IIb | ++ | 1.3 | 1/3 | 8/17 | n.k. | LFH |

[a]Serum autoantibodies: −/+=0.5–1; +=1–20; ++>20 nmol/L.
[b]n.k.=not known.
[c]Thymus histology: LFH=lymphoid follicular hyperplasia.
[d]LFH with cortical involution.

### ACKNOWLEDGMENTS

We thank Prof. Dr W. Nix (Mainz, FRG), Prof. Dr W. Mortier (Wuppertal, FRG) and Prof. Dr K. Stehr (Erlangen, FRG) for providing thymectomy specimens and clinical data. Furthermore, we thank Ms Ch. Kohaut and Ms A. Pietz and Mr E. Schmitt for expert technical assistance, and Drs R. Schoepfer and S. Heinemann for providing cDNA probes. HLA typing was kindly performed by Prof. E. Albert and Dr S. Scholz, Nationales Referenzlabor für Gewebetypisierung, Poliklinik, Universität München. Supported by grant Ki370/1-1 of the German Research Foundation (DFG) to A. M. and T. K.

*(Received May 14, 1991)*

*(Accepted September 25, 1991)*

### REFERENCES

Ausubel F.M., Brent R., Kingstone R.E., Moore D.D., Seidman J.G., Smith J.A., and Struhl K., Eds. (1987). Current protocols in molecular biology (New York: Greene Publishing Associates and John Wiley).

Boulter J.B., Goldman D., Evans K., Martin G., Stengelin S., Heinemann S., and Patrick J. (1986). Isolation, sequence and preparation of a cDNA clone coding for the gamma subunit of the mouse acetylcholine receptor. J. Neurosci. Res. **16:** 37–49.

Brenner H.R., Witzemann V., and Sakmann B. (1990). Imprinting of acetylcholine receptor messenger RNA accumulation in mammalian neuromuscular synapses. Nature **344:** 544–547.

Changeux J.P., and Revah F. (1987). The acetylcholine receptor molecule: Allosteric sites and the ion channel. TINS **10:** 245–250.

Chilosi M., Iannuci A., Fiore-Donati L., Tridente G., Pampanin M., Pizzolo M., Ritter M., Bofill M., and Janossy G. (1986). Myasthenia gravis: Immunological heterogeneity in microenvironmental organization of hyperplastic and neoplastic thymuses suggesting different mechanisms of tolerance breakdown. J. Neuroimmunol. **11:** 191–204.

Compston D.A., Vincent A., Newsom-Davis J., and Batchelor J.R. (1980). Clinical, pathological, HLA antigen, and immunological evidence for disease heterogeneity in myasthenia gravis. Brain **103:** 579–601.

Dipaola M., Czajowski C., and Karlin A. (1989). The sidedness of the COOH terminus of the acetylcholine receptor-delta-subunit. J. Biol. Chem. **264:** 15457–15463.

Drachman D.B., Adams R.N., Stanley E.F., and Pestronk A. (1980). Mechanisms of acetylcholine receptor loss in myasthenia gravis. J. Neurol. Neurosurg. Psychiatry **43:** 601–610.

Geuder K.I., Schoepfer R., Kirchner T., Marx A., and Müller-Hermelink H.K. (1989). The gene of the alpha-subunit of the acetylcholine receptor: Molecular organization and transcription in myasthenia-associated thymomas. Thymus **14:** 179–186.

Geuder K.I., Marx A., Schoepfer R., Witzemann V., Schalke B., Kirchner T., and Müller-Hermelink H.K. (1992). Genomic organisation and lack of transcription of the nicotinic acetylcholine receptor subunit genes in myasthenia gravis-associated thymoma. Lab. Invest. In press.

Giard D.J., Aaronson S.A., Todaro G.J., Arnstein P., Kersey J.H., Dosik H., and Parks W.P. (1973). In vitro cultivation of human tumors: Establishment of cell lines derived from a series of solid tumors. J. Natl. Cancer Inst. **51:** 1417–1423.

Hara Y., Ueno S., Uemichi T., Takahashi N., Yorifuji S., Fujii Y., and Tarui S. (1991). Neoplastic epithelial cells express alpha-subunit of muscle nicotinic acetylcholine receptor in thymoma from patients with myasthenia gravis. FEBS Lett. **279:** 137–140.

Kao I., and Drachman D.B. (1977). Thymus muscle cells bear acetylcholine receptors: Possible relation to myasthenia gravis. Science **195:** 74–75.

Kirchner T., Geuder K.I., Marx A., and Müller-Hermelink H.K. (1990). Detection of nicotinic acetylcholine receptors in tumours with rhabdomyomatous differentiation. Verh. Dtsch. Ges. Pathol. **74:** 409–414.

Kirchner T., Hoppe, F., Müller-Hermelink H.K., Schalke B., and Tzartos S. (1987). Acetylcholine receptor epitopes on epithelial cells of thymoma in myasthenia gravis. Lancet **1:** 218.

Kirchner T., Hoppe F., Schalke B., and Müller-Hermelink H.K. (1988b). Microenvironment of thymic cells in myasthenia gravis. Virchows Arch. (Cell Pathol.) **54:** 295–302.

Kirchner T., and Müller-Hermelink H.K. (1989). New approaches to the diagnosis of thymic epithelial tumors. *In:* Progress in surgical pathology, vol. 10, Fenoglio-Preiser, C.M., Wolff, M., Rilke, F., Eds. (Philadelphia: Field and Wood Inc.), pp. 167–186.

Kirchner T., Schalke B., Melms A., Kügelgen T., and Müller-Hermelink H.K. (1986). Immunohistochemical patterns of non-neoplastic changes in the thymus in myasthenia gravis. Virchows Arch. (Cell Pathol.) **52:** 237–257.

Kirchner T., Tzartos S., Hoppe F., Schalke B., Wekerle H., and Müller-Hermelink H.K. (1988a). Pathogenesis of myasthenia gravis: Acetylcholine receptor-related antigenic determinants in tumor-free thymuses and thymic epithelial tumors. Am. J. Pathol. **130:** 268–280.

Kobayashi T., Mishina M., Michikawa M., Kameda Y., Matsumoto Y., Tsukogoshi T., and Kamo I. (1990). Alpha-, beta-, gamma-, delta- and epsilon-subunit-specific mRNA of acetylcholine receptor (AchR) expressed in cultured rat thymus. J. Neurol. Sci. **98(Suppl.):** 229.

Kubalek E., Ralston S., Lindstrom J., and Unwin N. (1987). Location of subunits within the acetylcholine receptor by election image analysis of tubular crystals from Torpedo marmorata. J. Cell Biol. **105:** 9–18.

Luther M.A., Schoepfer R., Whiting P., Casey B., Blatt Y., Montal M.S., Montal M., and Lindstrom J. (1989). A muscle acetylcholine receptor is expressed in the human medulloblastoma cell line TE671. J. Neurosci. **9:** 1082–1096.

Marx A., Kirchner T., Hoppe F., O'Connor R., Schalke B., Tzartos S., and Müller-Hermelink H.K. (1989). Proteins with epitopes of the acetylcholine receptor in epithelial cell cultures of thymomas in myasthenia gravis. Am. J. Pathol **134:** 865–877.

Marx A., O'Connor R., Geuder K.I., Hoppe F., Schalke B., Tzartos S., Kalies I., Kirchner T., and Müller-Hermelink H.K. (1990). Characterization of a protein with an acetylcholine receptor epitope from myasthenia gravis-associated thymomas. Lab. Invest. **62:** 279–286.

Marx A., Osborn M., Tzartos S., Geuder K.I., Schalke B., Nix W., Kirchner T., and Müller-Hermelink H.K. (1992). A striational muscle antigen and myasthenia gravis-associated thymomas share an acetylcholine receptor epitope. Dev. Immunol. In press.

Mishina M., Takai T., Imoto K., Noda M., Takahashi T., Numa S., Methfessel C., and Sakmann B. (1986). Molecular distinction between fetal and adult forms of muscle acetylcholine receptor. Nature **321:** 406–411.

Müller-Hermelink H.K., Marino M., and Palestro G. (1986).

The human thymus: Histophysiology and pathology. Current topics in pathology, vol. 75, Müller-Hermelink, Ed. (Berlin: Springer).

Nelson S., and Conti-Tronconi B.M. (1990). Adult thymus expresses an embryonic nicotinic acetylcholine receptor-like protein. J. Neuroimmunol. **29:** 81–92.

Osserman K.E. (1958). Myasthenia gravis. (New York: Grune & Stratton Inc.).

Patrick J., Boulter J., Goldman D., Gardner P., and Heinemann S. (1987). Molecular biology of the nicotinic acetylcholiner receptor. Ann. N.Y. Acad. Sci. **505:** 194–207.

Schluep M., Willcox N., Vincent A., Dhoot G.K., and Newsom-Davis J. (1987). Acetylcholine receptors in human thymic myoid cells in situ: An immunohistological study. Ann. Neurol. **22:** 212–220.

Schoepfer R., Luther M., and Lindstrom J. (1988). The human medulloblastoma cell line TE671 expresses a muscle-like acetylcholine receptor: Cloning of the alpha-subunit cDNA. FEBS Lett. **226:** 235–240.

Siara J., Rüdel R., and Marx A. (1991). Absence of acetylcholine-induced current in epithelial cells from thymus glands and thymomas in myasthenia gravis patients. Neurology **41:** 128–131.

Tzartos S., Langeberg L., Hochschwender S., Swanson L.W., and Lindstrom J. (1986). Characteristics of monoclonal antibodies to denatured Torpedo and to calf acetylcholine receptors: Species, subunit and region specificity. J. Neuroimmunol. **10:** 235–253.

Van de Velde R.L., and Friedman N.B. (1960). The thymic "myoidzellen" and myasthenia gravis. JAMA **198:** 287–288.

Venet-der Garabedian B., Bustarret F., Eymard B., Bach J.F., and Morel E. (1988). Myasthenia gravis sera and maturation of the acetylcholine receptor at developing neuromuscular junctions in the rat. 3[e] Colloque National sur les Maladies Neuromusculaires, Bordeaux. Resume des Communications, p. 223.

Vincent A., Whiting P., Schluep M., Heidenreich F., Lang B., Roberts A., Willcox N., and Newsom-Davis J. (1987). Antibody heterogeneity and specificity in myasthenia gravis. Ann. N.Y. Acad. Sci. **505:** 106–120.

Wekerle H., Ketelsen U.P., Zurn A.D., and Fulpius B.W. (1978). Intrathymic pathogenesis of myasthenia gravis: Transient expression of acetylcholine receptors on thymus derived myogenic cells. Eur. J. Immunol. **8:** 579–581.

Weinberg C.B., and Hall Z.W. (1979). Antibodies from patients with myasthenia gravis recognize determinants unique to extrajunctional receptors. Proc. Natl. Acad. Sci. USA **76:** 504–508.

Witzemann V., Barg B., Criado M., Stein E., and Sakmann B. (1989). Developmental regulation of five subunit specific mRNAs encoding acetylcholine receptor subtypes in rat muscle. FEBS Lett. **242:** 419–424.

Witzemann V., Barg B., Nishikawa Y., Sakmann B., and Numa S. (1987). Differential regulation of muscle acetylcholine receptor gamma and epsilon-subunit mRNAs. FEBS Lett. **223:** 104–112.

Witzemann V., Stein E., Barg B., Konno T., Koenen M., Kues W., Criado M., Hoffmann M., and Sakmann B. (1990). Primary structure and functional expression of the alpha-, beta-, gamma-, delta- and epsilon-subunits of the acetylcholine receptor from rat muscle. Eur. J. Biochem. **194:** 437–448.

